# Alcohol policy compliance among retailers in Bhutan: a multisite community intervention study

**DOI:** 10.1186/s12889-021-11932-0

**Published:** 2021-10-19

**Authors:** Tshewang Gyeltshen, Tshering Penjor, Lham Dorji, Lobzang Tshering, Kinley Dorji, Bhim Nath Subedi, Dorji Tshering, Yvonne Yiru Xu, Gampo Dorji, Ghislain Nono Gueye

**Affiliations:** 1 Tsirang Hospital, Damphu, Tsirang District Bhutan; 2 Tsirang District Administration, Health Sector, Damphu, Tsirang Bhutan; 3 District Administration, Health Sector, Pema Gatshel, Bhutan; 4 Gelephu Central Regional Referral Hospital, Gelephu, Bhutan; 5 WHO, SEARO, New Delhi, India; 6 Louisiana Tech University, Ruston, Louisiana USA

**Keywords:** Alcohol, Bhutan, Compliance, Proxy purchase

## Abstract

**Background:**

Alcohol use is a major public health problem in Bhutan. Compliance with regulations at the point of sale is an important strategy in alcohol control. Retail outlets were briefed on sale regulations and provided notification of rules, which they were directed to display on the premises. The extent to which licensed alcohol outlets responded to possible alcohol purchases was assessed through the use of young proxy-purchasers, adults feigning alcohol intoxication and sober adults. A total of 854 visits (pre versus post visits) were made across four district towns. Two towns (Damphu town in Tsirang district and Pema Gatshel town in Pema Gatshel district) received pre- and post-intervention purchase surveys, while the other two neighbouring towns (Khuruthang town in Punakha and Bajo town in Wangdue) were administered only baseline surveys.

**Method:**

We used a pre- and post-test community intervention design covering all alcohol retailers both on premise (bar, hotel, restaurant, karaoke bars) and off premise (grocery shops). Compliance with alcohol regulations at the point of sale was assessed through the use of young proxy-purchasers, adults feigning alcohol intoxication and sober adults.

**Results:**

Retailers rarely checked the age and/or identification (ID) of the proxy-purchasers before the intervention. There was a 22.7% (8.6, 37) percent increase in compliance with laws after the intervention. While some strategies are suggested, the strongest and most effective measure to prevent under-age drinking and service to intoxicated individuals is more rigorous enforcement of existing liquor laws.

**Conclusion:**

Alcohol control requires ongoing government enforcements, supplemented by public awareness and knowledge.

## Background

Alcohol use is a major public health problem globally. In Bhutan, alcohol-related liver disease is the number one cause of mortality, accounting for 11% of the total deaths that occurred in health facilities in 2019 [1]. Mortality is even higher after accounting for alcohol-related deaths due to heart disease, stroke, injuries, suicides and road traffic crashes.

Drinking is pervasive in Bhutanese society, with most drinkers starting at an early age. One-third (33.1%) of adult Bhutanese are current drinkers (last 30 days) [2], which is higher than figures in neighbouring countries (Nepal 18%, Myanmar 20% and Sri Lanka 18% and India 11%). Alcohol consumption is also highly prevalent among young people. A Bhutan school-based survey conducted in 2016 found that 24% of Bhutanese students below 18 years of age were current drinkers compared to their counterparts in Nepal (5.5%), Myanmar (4.7%) and Sri Lanka (3.2%) [3]. Among underage students, 16.2% of females were current users of alcohol in Bhutan, contrary to other countries in Southeast Asia, where consumption among females is negligible [4]. Nearly 17% of the Bhutanese population (half of all current drinkers) are heavy episodic drinkers [5]. Four percent of the population reported needing their first drink in the morning, indicating alcohol dependence [5]. In addition to ill health, excessive drinking has significant social and economic consequences [6].

Bhutan has a comprehensive set of alcohol laws. These include restriction of sales and supply to those under the age of 18, restriction of sales to alcohol-intoxicated individuals, prohibition of alcohol sales before 1 p.m. and after 10 p.m., alcohol sales prohibited on Tuesdays and drink driving regulated at a blood alcohol level of 0.8%, or 0% for commercial drivers [7]. Apart from the drink driving laws, alcohol sale regulations are perceived to be poorly enforced. This allows alcohol to be easily accessible during non-legal hours of the day regardless of the rules. A study conducted in 2013 by Dorji et al. documented the widespread breach of practices in the sale and supply of alcohol on premises in the capital city of Thimphu [8].

Educating alcohol retailers on the alcohol sales requirements and legal consequences of violations combined with the presence of enforcement is known to improve retailers’ compliance [8]. Earlier evidence relating to alcohol sales and compliance by retailers differs according to the type of intervention provided. The compliance of retailers was found to be improved for age restrictions on alcohol sales when positive feedback through letters was provided in the Netherlands [9]. Community-based studies in Australia, Stockholm and the UK have shown that social marketing, community mobilization and community-based interventions are effective in reducing youth access to alcohol [10–12]. Another study in the United Kingdom documented that alcohol service refusal to pseudo-intoxicated buyers decreased with server training and community mobilization [13].

This study aimed to [1] assess current levels of compliance with alcohol sale regulations in four townships in Bhutan, [2] evaluate the effect of alcohol legal information education and enforcement intervention, and [3] assess factors associated with compliance with legal alcohol sales amongst licensed retailers.

## Methodology

### Evaluation design

We used a pre- and post-test community intervention design covering all alcohol retailers both on premise (bar, hotel, restaurant, karaoke bars) and off premise (grocery shops). All establishments that had alcohol sales permits were selected from four district towns: Pema Gatshel town in Pema Gatshel district in the east, Damphu town in Tsirang district in the central region and adjoining Khuruthang town in Punakha district and Bajo town in Wangdue district in the western region. The four districts were selected to represent the three geographical administrative regions commonly used as a reference in the country. These four districts are also similar in terms of demography and socioeconomic status. Pemagatshel and Tsirang districts have total populations of 23,799 and 23,771, respectively, while Wangdue has 46,585 and Punakha has 30,790 [14]. Due to limited resources to implement the survey, only two districts with similar demographics were selected to administer the post-intervention survey.

### Proxy purchasers

Outlets’ willingness to sell alcohol was assessed using proxy purchasers comprising underage-looking purchasers who were actually more than eighteen years old (> 18 years), sober adults and adults acting alcohol-intoxicated. Four underage-looking young adults (2 females and 2 males) and 4 adults (2 females and 2 males) were recruited in Damphu town. In Pema Gatshel town, six purchasers – two underage-looking young adults (2 females and 2 males) who were both more than 18 years old and 4 adults (2 females and 2 males) – were recruited. In Bajo and Khuruthang towns, eight underage-looking purchasers (2 females and 6 males) (> 18 years) and eight adult purchasers were recruited. Purchasers were recruited based on their acting skills. Underage-looking young adults were identified as ‘below 18 years’ by a group of judges. These proxy purchasers were trained on survey instruments using real-time purchasing scenarios. The duration of training varied from 2 days in Damphu town to 3 h of training in Pema Gatshel and 1 day for Bajo and Khuruthang towns. The same purchasers completed the purchase surveys in Bajo and Khuruthang. Separate groups of purchasers were recruited in pre- and post-intervention purchase surveys in intervention districts of Pema Gatshel town and Damphu town.

The proxy clients attempted purchase at illegal hours (before 1 pm during nondry days), during dry days (Tuesday) and during legal hours (1–10 pm on a nondry day) for underage-looking and pseudo-intoxicated adults. Legal visits between 1 and 10 p.m. by sober adults were included for comparison too. Every purchase attempt was made by a pair of proxy clients. After leaving the establishment, the purchasers were required to report to an enumerator who awaited at a distance. The enumerator filled out the survey tool as reported by the purchasers in their presence. Pre-intervention purchases were conducted in August 2019, and post-intervention purchase surveys were conducted in March 2020. Only pre-intervention purchase surveys were completed in Bajo and Khuruthang for baseline data acquisition.

### Intervention

The intervention consisted of a briefing of outlet owners and sellers explaining the alcohol sales rules and warning of the legal consequences of breach of alcohol rules by government officers. The briefing was led by the *Dzongdag (*district governor), police officer, trade officer and district health officer. One representative from each retail outlet was invited from the study sites. The briefing was conducted on 29 October 2019 in Damphu and 10 October 2019 in Pema Gatshel town. Briefing included existing alcohol sales rules, penalties and possible charges for violators and the negative health effects of alcohol. The alcohol regulation toolkit containing information on sales rules and a copy of the laminated A3-size alcohol rule notification was handed over by the governor, and officials and sellers were asked to display the notification in a prominent location within the premise. Retailers were informed that surprise checks would be conducted, and violators penalized as per the regulations, which involve court hearings if required. The intervention was well received, with all vendors agreeing that there was common responsibility for responsible alcohol sales. All vendors embraced the alcohol rules and policies, some of which they were not aware of previously. At the end of the intervention, all vendors agreed that they would be compliant with the alcohol rules and regulations as briefed during the day.

### Ethics approval

Ethics approval was obtained from the Research and Ethics Board of the Ministry of Health, Royal Government of Bhutan for purchase surveys in Damphu and Pema Gatshel towns, and local administrative approvals were obtained for Tsirang and Pema Gatshel district administrations. Wangdue and Punakha purchase attempts were performed as a part of the routine monitoring activity of the chief medical officer at Bajo Hospital, and no ethical approval was sought.

### Sampling

All establishments with alcohol sales licences (i.e., bars, hotels, restaurants, grocery, karaoke bars) in Damphu and Pema Gatshel towns and Bajo and Khuruthang were included in the study. A total of 60 outlets in Damphu and 12 outlets in Pema Gatshel town were provided with interventions, while 55 outlets in Bajo and Khuruthang were included in the purchase survey (Table 1.)

**Table 1:** Number of purchases made by shopper type and time/day of purchase; alcohol policy compliance among retailers in Bhutan, 2019–2020

		Damphu town, Tsirang				Pema Gatshel town, Pema Gatshel				Wangdue (Bajo) & Punakha (Khuruthang)			
		Time/day of purchase attempt				Time/day of purchase attempt				Time/day of purchase attempt			
**Intervention**	**Shopper type**	before 1 p.m.	1–10 p.m.	Tuesday	**Total**	before 1 p.m.	1–10 p.m.	Tuesday	**Total**	before 1 p.m.	1–10 p.m.	Tuesday	**Total**
**Pre-**	*Sober adults*	53	0	40	93	5	7	10	22	–	3	103	106
	*Intoxicated adults*	4	54	0	58	0	12	0	12	–	36	–	36
	*Underage*	7	51	0	58	0	11	0	11	–	49	29	78
	***Total***	64	105	0	209	5	30	0	45	–	88	132	220
**Post-**	*Sober adults*	24	24	49	97	24	24	49	97	–	–	–	–
	*intoxicated adults*	1	52	1	54	1	52	1	54	–	–	–	–
	*Underage*	0	24	15	39	0	24	15	39	–	–	–	–
	***Total***	25	100	65	190	25	100	65	190	–	–	–	–

### Analysis

The data were entered and managed using Epidata Entry Software version 3.1. A double entry was made and validated. Data analysis was carried out using Stata 15 IC (StataCorp. 2017. *Stata Statistical Software: Release 15*. College Station, TX: StataCorp LLC). The graphics were generated using open software R, (R Core Team (2020). R: A language and environment for statistical computing. R Foundation for Statistical Computing, Vienna, Austria.). Analysis included all completed purchase attempts, which included illegal purchasing of alcohol in 4 different scenarios: [1] before 1 p.m., [2] on Tuesdays, [3] to underage-appearing patrons (i.e., younger than 18 years old), and [4] to patrons who appeared to be intoxicated. While alcohol purchasing after 11 p.m. would be illegal, these purchasing attempts were not carried out as none of the outlets were open until 11 p.m. A few purchase attempts by sober adults during legal hours were also completed for comparison.

Information on the main characteristics of the sampled establishments was summarized. The primary outcome was compliance defined as failed purchase of illegal alcohol sales. The effect of the intervention on the difference in compliance rate with legal restrictions was estimated using a linear probability regression model. Both crude effects and adjusted effects were estimated for the full sample in Tsirang and Pema Gatshel. Effects were estimated for each purchaser scenario, and a two-way cluster was accounted for in the standard error at the level of the shopper and the establishment using Stata command ivreg2. To account for overlapping subgroups, (i.e., underaged shopper on Tuesday), each shopper type and time/day of attempt were included as covariates for each subgroup analysis. Finally, we identified predictors of compliance with alcohol service laws using a multivariable logistic model. Our models were ranked according to their goodness of fit using a stepwise forward Akaike information criterion (AIC) method. All variables in the survey were included in the model selection process, as they are known factors associated with alcohol sales based on authors’ knowledge of the literature. Variables were grouped into categories and added by category, which included server information, smoking environment, nature of purchase, venue condition and signage information. Each model was tested, and the final model was selected based on the lowest AIC score.

## Results

When comparing pre- and post-interventions, there was an increase in establishments that displayed signage showing alcohol regulations, an increase in those that provided a prominent placement and even an increase in the display of smoking prohibition signage (Table 2). The characteristics of customer volume, servers, type, age and gender remained comparable between the two purchase surveys.

**Table 2:** Characteristics of completed mystery shopper purchase attempts (Damphu and Pema Gatshel); alcohol policy compliance among retailers in Bhutan, 2019–2020

	Pre-intervention (%)		Post-intervention (%)	
	n	%	N	%
**Total**	**254**	**–**	**234**	**–**
**Outlet type**				
Bar/Karaoke Bars	81	31.9	91	38.9
Hotel/Lodge	30	11.8	13	5.6
Restaurant	79	31.1	59	25.2
Grocery	48	18.9	52	22.2
Others	16	6.3	19	8.1
**Overall condition of establishment**				
Good	8	31.5	51	21.8
Fair	149	58.7	179	76.5
Poor	25	9.8	4	1.7
**Lighting of establishment**				
Poorly lit	22	8.7	8	3.4
Fairly Lit	163	64.2	190	81.2
Well Lit	69	27.2	36	15.4
**Server type**				
Manager/owner	235	95.1	188	91.3
Waiter	12	4.9	18	8.7
**Customer flow of establishment**				
Idle	128	50.4	124	53.0
Engaged but not busy	100	39.3	83	35.5
Very busy	26	10.2	27	11.5
**Server position**				
Manager/Owner	242	95.3	214	91.5
Waiter	12	4.7	20	8.6
**Server gender**				
Male	74	29.1	55	23.5
Female	180	70.9	179	76.5
**Approximate server age**				
Above 30 years	200	78.7	182	77.8
Less than 30 years	54	21.3	52	22.2
**Alcohol beverage asked for**				
Beer	145	57.1	151	64.5
Hard Drinks	7	31.1	40	17.1
Other	30	11.8	43	18.4
**Signage/Information on alcohol regulations**				
No	232	91.3	102	43.6
Yes	22	8.7	132	56.4
**Signage information displayed prominently**				
No	50	27.8	9	4.6
Yes	130	72.2	189	95.5
**Placement of signage was prominent**				
No	50	27.8	12	5.4
Yes	130	72.2	210	94.6
**Establishment displayed smoking prohibition signage**				
No	115	45.3	17	7.3
Yes	139	54.7	217	92.7

Failed purchase attempts (i.e., compliance with legal regulations) prior to intervention were poorest in Pema Gatshel town, where all purchasing attempts were successful, and compliance was highest in Damphu town at 19.6%. Compliance with restriction of sales on Tuesday was relatively higher than compliance in other types of scenarios, and the lowest compliance was seen with intoxicated patrons (Table 3).

**Table 3:** Pre-intervention compliance rate or alcohol purchasing by region; alcohol policy compliance among retailers in Bhutan, 2019–2020

Region	Damphu, Tsirang		Pema Gatshel		Bajo/Khuruthang		Total	
	Purchase attempts	Proportion refused	Purchase attempts	Proportion refused	Purchase attempts	Proportion refused	Purchase attempts	Proportion refused
**Type of purchase**								
Before 1 p.m.	64	17.2%	5	0.0%	2	50.0%	71	16.9%
Tuesday	40	30.0%	10	0.0%	132	28.8%	182	27.5%
Underage-appearing patrons	58	32.8%	11	0.0%	78	2.6%	147	14.3%
Pseudo-intoxicated patrons	58	5.2%	12	0.0%	36	2.8%	106	3.8%
Full sample	209	19.6%	38	0.0%	217	18.9%	474	17.3%

**Fig. 1:**
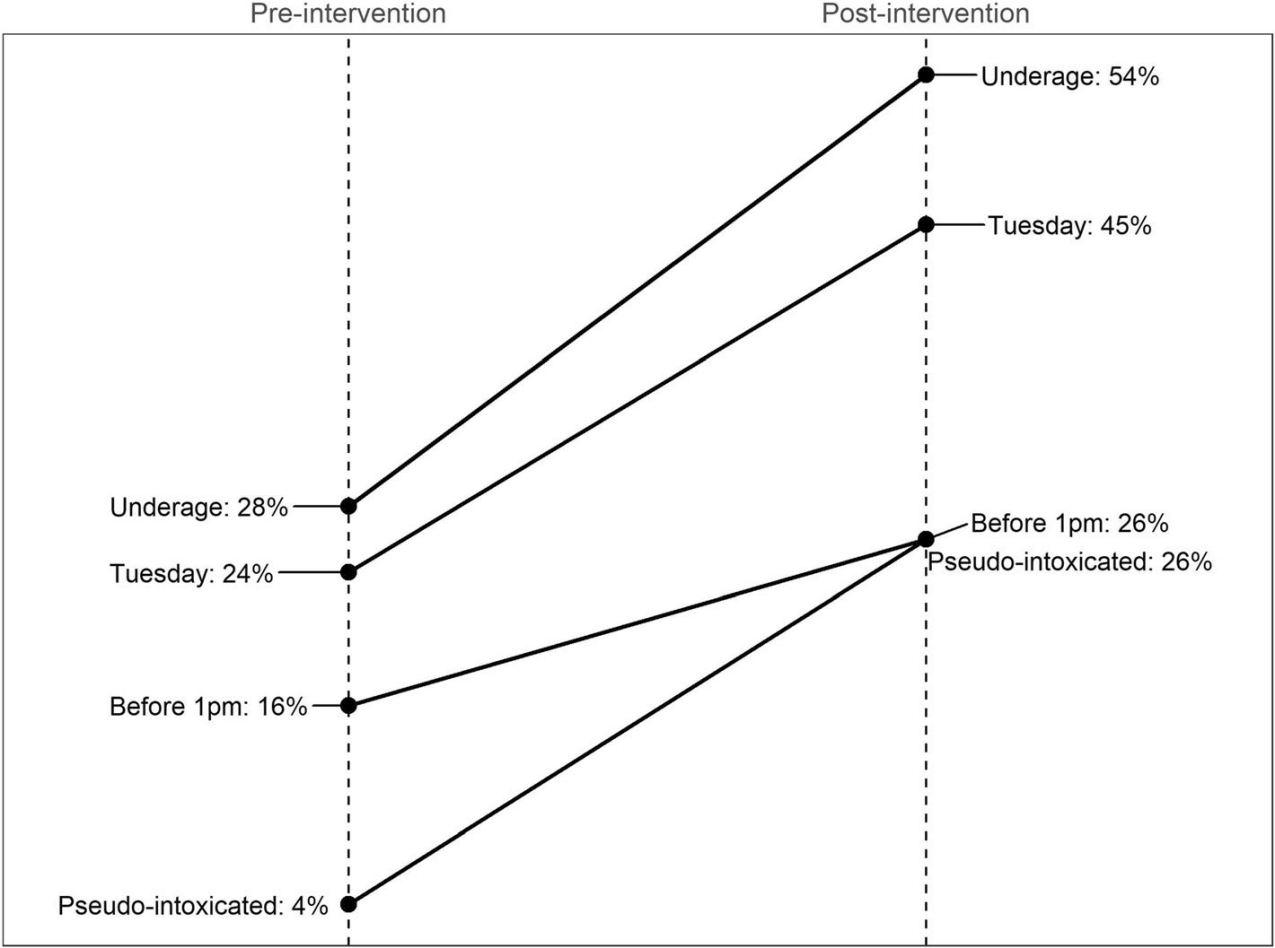
Impact of intervention on the compliance rate by purchase type; alcohol policy compliance among retailers in Bhutan, 2019–2020

**Fig. 2:**
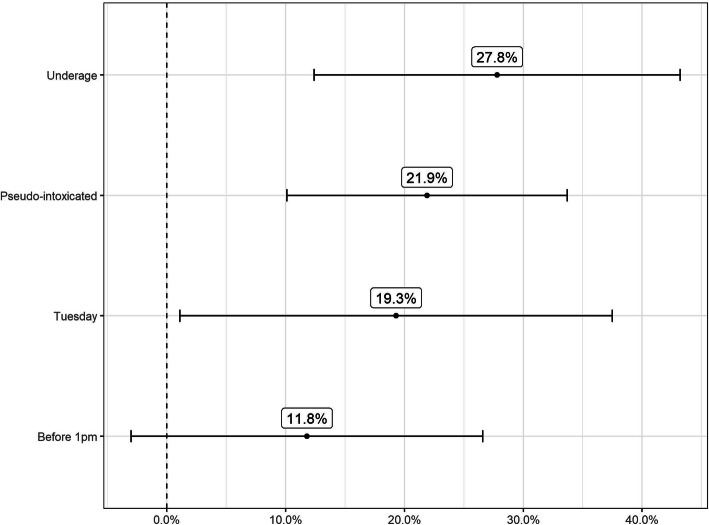
Adjusted effect estimates and 95% CI of intervention on the compliance rate by purchase type; alcohol policy compliance among retailers in Bhutan, 2019–2020

There was a statistically significant increase in refusal of alcohol purchase attempts by 22.7% overall and in all purchasing scenarios except for illegal purchasing before 1 pm. When disaggregated by purchasing scenario, the increase in refusal was highest in the underage-appearing patron scenario. The same relationship remains after adjusting for other purchasing scenarios (Table 4). The change in the compliance rate for each purchase category and the adjusted effect estimates with a 95% confidence interval are displayed in Fig. 1 and Fig. 2, respectively.

**Table 4:** Compliance rate by the type of purchase and effect estimates (Tsirang and Pema Gatshel); alcohol policy compliance among retailers in Bhutan, 2019–2020

Type of purchase	Pre-intervention		Post-intervention		Unadjusted effect estimates		Adjusted effect estimates	
	Purchase attempts	Proportion refused	Purchase attempts	Proportion refused	Difference	95% CI	Difference	95% CI
Before 1 p.m.	69	16%	34	26%	10.5%	(−6.4, 27.5%)	11.8%	(−3.0, 26.6%)
Tuesday	50	24%	76	45%	20.7%	(2.8, 39.7%)	19.3%	(1.1, 37.5%)
Underage-appearing patrons	69	28%	50	54%	26.5%	(12.0, 40.9%)	27.8%	(12.4, 43.2%)
Pseudo-intoxicated patrons	70	4%	65	26%	21.9%	(10.8, 33.0%)	21.9%	(10.1, 33.7%)
Full sample	**247**	**17%**	**206**	**38%**	22.7%	(8.6, 37.0%)	–	–

**Fig. 3:**
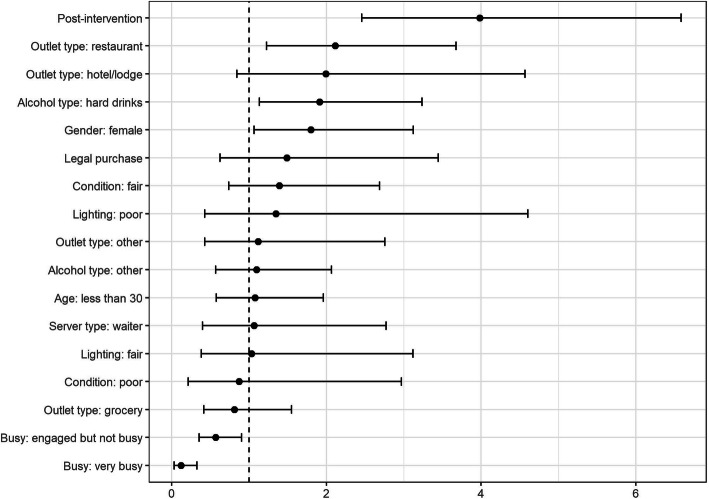
Model 1 odds ratio estimates and 95% confidence intervals; alcohol policy compliance among retailers in Bhutan, 2019–2020

**Fig. 4:**
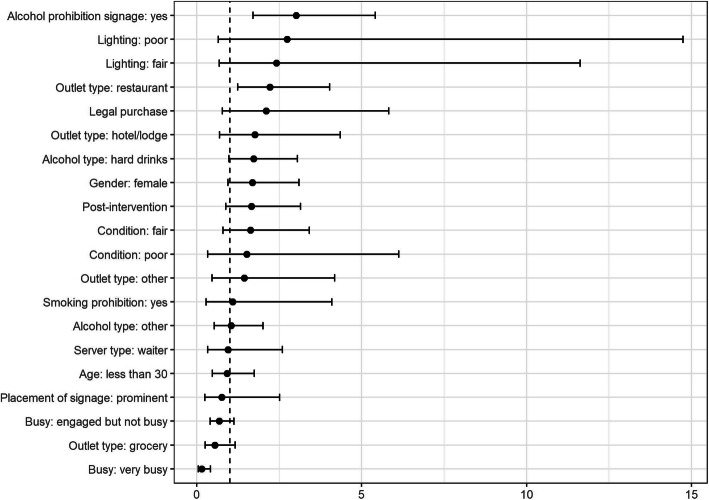
Model 2 odds ratio estimates and 95% confidence intervals; alcohol policy compliance among retailers in Bhutan, 2019–2020

Table 5 shows the adjusted odds ratios of the predictors that are associated with refusal of purchase attempts in the two models. The receipt of intervention was a statistically significant predictor for purchase refusal in model 1. This relationship was not significant when adjusted for establishments that displayed signage on alcohol prohibition provided during the intervention. Establishments where any type of signage relating to alcohol prohibition was displayed were 3.0 times more likely to refuse alcohol sales (OR: 3.01, 95% CI: 1.67–5.36). Restaurants were twice as likely as bars/karaoke bars to comply with alcohol sales restrictions (OR: 2.22, 95% CI 1.23–3.99). Establishments that were very busy were significantly less likely to comply with prohibition than those that were idle (OR: 0.15, 95% CI: 0.05–0.46). Figures 3 and 4 are forest plots of the odds ratios with 95% confidence intervals for models 1 and 2, respectively.

**Table 5:** Predictors for compliance with alcohol purchasing regulations; alcohol policy compliance among retailers in Bhutan, 2019–2020

	Model 1					Model 2				
	OR	SE	p- value	95% CI		OR	SE	***p***-value	95% CI	
**Post-intervention (compared to pre)**	3.98	1.00	0.00	2.44	6.51	1.66	0.54	0.12	0.87	3.13
**Legal purchase**	1.49	0.64	0.36	0.64	3.48	2.10	1.08	0.15	0.77	5.73
**Outlet type**										
Bars/Karaoke	1.00	–	–	–	–	1.00	–	–	–	–
Hotel/lodge	1.99	0.85	0.11	0.86	4.62	1.76	0.82	0.23	0.70	4.38
Restaurant	2.12	0.59	0.01	1.22	3.66	2.22	0.66	0.01	1.23	3.99
Grocery	0.81	0.27	0.53	0.42	1.56	0.55	0.21	0.13	0.26	1.18
Other	1.12	0.53	0.81	0.44	2.82	1.44	0.80	0.52	0.48	4.26
**Server position**										
Manager/Owner	1.00	–	–	–	–	1.00	–	–	–	–
Waiter	1.07	0.52	0.90	0.41	2.79	0.94	0.49	0.91	0.34	2.62
**Sex of server**										
Male	1.00	–	–	–	–	1.00	–	–	–	–
Female	1.80	0.49	0.03	1.05	3.08	1.69	0.51	0.08	0.94	3.05
**Presumed age of the server**										
30 and over	1.00	–	–	–	–	1.00	–	–	–	–
Less than 30	1.08	0.33	0.81	0.59	1.98	0.92	0.31	0.79	0.48	1.76
**Alcohol beverage asked for**										
Beer	1.00	–	–	–	–	1.00	–	–	–	–
Hard Drinks	1.91	0.51	0.02	1.13	3.23	1.72	0.50	0.06	0.97	3.04
Other	1.10	0.36	0.77	0.58	2.09	1.04	0.35	0.90	0.54	2.03
**Customer flow of establishment**										
Idle	1.00	–	–	–	–	1.00	–	–	–	–
Engaged but not busy	0.57	0.14	0.02	0.36	0.91	0.68	0.18	0.13	0.40	1.13
Very busy	0.12	0.07	0.00	0.04	0.36	0.15	0.09	0.00	0.05	0.46
**General condition of establishment**										
Good	1.00	–	–	–	–	1.00				
Fair	1.39	0.46	0.32	0.73	2.65	1.63	0.60	0.19	0.79	3.36
Poor	0.87	0.57	0.84	0.24	3.17	1.52	1.11	0.57	0.36	6.37
**Lighting of establishment**										
Poorly lit	1.00	–	–	–	–	1.00	–	–	–	–
Fairly Lit	1.03	0.54	0.95	0.37	2.90	2.41	1.70	0.21	0.61	9.60
Well Lit	1.35	0.81	0.62	0.42	4.35	2.74	2.12	0.20	0.60	12.52
**Establishment displayed any type of alcohol prohibition signage**										
No	–	–	–	–	–	1.00	–	–	–	–
Yes	–	–	–	–	–	3.01	0.89	0.00	1.69	5.36
**Placement of signage was prominent**										
No	–	–	–	–	–	1.00	–	–	–	–
Yes	–	–	–	–	–	0.75	0.44	0.63	0.24	2.38
**Establishment displayed smoking prohibition signage**										
No	–	–	–	–	–	1.00	–	–	–	–
Yes	–	–	–	–	–	1.08	0.73	0.91	0.29	4.09

## Discussion

To date, this is the first multisite study conducted in Bhutan to assess alcohol retail establishments’ compliance with alcohol sale regulations. We applied educational interventions to improve policy knowledge amongst retailers through organized training sessions. The baseline refusal rate in the four districts was very low (17.3%), which indicates poor compliance with alcohol sales rules and regulations at the point of sales. The compliance with Tuesdays (27.5%) as dry days was most noticeable, while sales to intoxicated adults (3.8%) were least compliant, suggesting a possible lack of knowledge on alcohol regulations. This gap was largely improved post-intervention, which suggests that educational intervention and enforcement portrayals were effective. More concerning, compliance with denying sales to underaged purchasers was only 14.3% at baseline. This would probably explain the high prevalence of alcohol use in Bhutan amongst those under 18 years of age [3].

Of the four districts with baseline comparisons, Pema Gatshel performed worst in terms of its baseline refusal rate (0.0%), although due to the low number of purchase attempts, it is difficult to be conclusive. Damphu performed well in refusal to underaged patrons compared to other regions (32.8%). Drinking is pervasive and more common among the eastern districts of Bhutan [15]. This significant variation in baseline compliance of the eastern district (Pema Gatshel) and western-central districts (Punakha, Wangdue and Tsirang) can be explained by this phenomenon. The intervention was effective in improving the overall refusal rate, with the most prominent improvements for underage patrons (53.9%). No effects of the interventions were seen for restrictions of alcohol sales before 1 p.m. which may reflect implementation and pragmatic challenges for compliance by establishments. The establishments are found busy at almost all timestime of day, and it is simply easier to serve drinks than to wait for the allowed time. On any given day, the bar shelves with alcohol liquors would be open except for Tuesdays. We speculate that it is simply easier to refuse customers on an entire day, such as Tuesday, rather than restricting them to specific hours in a day. Further qualitative data may be helpful to better understand how such policies influence sellers’ behaviours.

Our analysis shows that displaying signage regarding alcohol prohibition in the store was predictive of compliance. There may be two explanations for this finding. First, this could suggest that requiring signage placement is an effective policy tool to ensure legal compliance with alcohol restrictions and should be made mandatory with sufficient enforcement. Second, the association noted here may be a proxy for the diligence and compliance of the establishment itself rather than due to the visible signalling of signage. We are unable to draw conclusions here, as we cannot draw causation from the display of signage based on our analysis.

The finding that busier establishments are less likely to comply is concerning. This suggests an underlying lack of diligence where the importance of complying with alcohol restriction is overlooked or not at the forefront of the minds of the server. Police enforcement and checks may resolve this concern. If owners are aware that random checks can be made and they would suffer repercussions such as paying a fine, then even amidst busy service times, legal compliance with the law comes to the forefront of one’s mind. Police enforcement should also consider conducting checks at peak hours. These findings are consistent with an earlier study performed in Thimphu, the capital city of Bhutan [16].

Compared to a previous study [8], this study had a larger reduction according to the intervention effect. However, absolute compliance post-intervention remained low at 38% compared to 34% in the last study, which also had an enforcement component. The post-intervention change is lower than we anticipated. Some of the study interventions coincided with the early phase of the COVID-19 pandemic in 2020. The enforcement visits were not conducted per protocol as initially designed, which may explain the lower effect. This indicates that legal education and training alone will not reach optimal levels of legal compliance. There may be gaps in educational training methods that could improve sellers’ awareness and room for increasing legal repercussions, such as increasing fines and penalties.

Bhutan, which has abundant access to alcohol [17] that is freely available at both off premise and on premise outlets, needs strong law enforcement. The alcohol laws are being violated without regard, as evidenced by our findings and previous findings. Substantial gaps remain in improving legal compliance with alcohol laws in Bhutan.

Future research should focus on evaluating the effectiveness of different components of education interventions in terms of their content as well as hand-out materials such as signage. Established perceptions of alcohol regulation should be better researched to understand potential drivers and barriers to compliance at the point of sale.

Our study also has several limitations. The study is subject to concerns about ecological fallacy. We did not have any control district to adjust for possible external factors that may explain the increase in compliance with alcohol regulations. Therefore, we cannot state that the increase in compliance was solely caused by the education interventions themselves. Second, the sample size was relatively small compared to other studies. However, Bhutan is not a densely populated country, and thus, what is sampled is inclusive of all establishments within selected district towns. Finally, our study evaluated the effects 5 months post-intervention. Without sustained enforcement and education, the effects may dissipate over time.

## Conclusion

Alcohol control requires ongoing government enforcement, supplemented by public awareness and knowledge. Our study demonstrated that despite long-standing alcohol sale regulations in Bhutan, compliance at the point of sale was low across all districts. The effectiveness of the policy of alcohol sales restriction before 1 p.m. is low and warrants further review or replacement with more effective alternatives. Moreover, education activities should be conducted periodically and possibly should be included as part of alcohol sales permit renewal requirements to ensure establishment awareness over time and sustained effects of the intervention. Furthermore, if local law enforcement wants to conduct random compliance checks, surveillance during peak alcohol sales hours would be appropriate. Current poor compliance not only results in large socioeconomic costs but also risks the future of the most vulnerable portion of the population – children and adolescents.

## Data Availability

Data and materials available from the corresponding author upon request.
